# The Effects of Interprofessional Education in Mental Health Practice: Findings from a Systematic Review

**DOI:** 10.1007/s40596-018-0951-1

**Published:** 2018-07-11

**Authors:** Michael Marcussen, Birgitte Nørgaard, Sidse Arnfred

**Affiliations:** 1 University of Copenhagen, Copenhagen, Denmark; 2 University of Southern Denmark, Odense, Denmark

**Keywords:** Interprofessional education, Undergraduate education, Clinical training in mental health, Review

## Abstract

**Objective:**

The aim of this study was to conduct a systematic review of studies describing the effects of interprofessional education (IPE) on undergraduate healthcare students’ educational outcomes, compared with conventional clinical training in mental health.

**Methods:**

MEDLINE, CINAHL, PsychINFO, and EMBASE were searched for studies published in January 2001–August 2017. All retrieved papers were assessed for methodological quality; Kirkpatrick’s model was employed to analyze and synthesize the included studies. The following search terms were used: undergraduate, interprofessional education, and educational outcomes.

**Results:**

The eight studies that met the inclusion criteria were highly diverse regarding the studied IPE interventions, methods, and outcomes. Participants included students receiving clinical training in mental health from the following professions: medicine, nursing, occupational therapy, physiotherapy, psychology, and social work. The results of the studies suggest that students respond well to IPE in terms of more positive attitudes toward other professions and improvement in knowledge and collaborative skills. Limited evidence of changes in behavior, organizational practice, and benefits to patients was found.

**Conclusion:**

Based on the eight included studies, IPE interventions appear to have an impact regarding positive attitudes toward other professions and increased knowledge of and skills in collaboration compared to conventional clinical training. However, further study of both the processes and the long-term impacts of undergraduate IPE in mental health is needed. The authors recommend that service users are involved in the implementation and evaluation of IPE interventions in mental health to undergraduate healthcare students.

Persons with mental illness often have complex needs whose effective care requires participation from a diversity of healthcare professionals [1–3]. Interprofessional collaboration has been promoted as an effective avenue to enhance the delivery of patient care [4–6]. However, the challenges of ensuring collaboration among team members in mental health are well testified [3, 7]. Interprofessional collaboration in the field is thus hampered by strong uniprofessional cultures, a diversity of approaches to the care and treatment of patients, and conflict over leadership [3, 7–9]. Interprofessional education (IPE) nevertheless continues to be invoked by policymakers as an effective method to improve collaboration [4, 6], and calls for its wider implementation across educational and clinical settings are frequently heard [10–12]. The World Health Organization (WHO) defines IPE: “(…) students from two or more professions learn[ing] about, from and with each other to enable effective collaboration and improve health outcomes” [4]*.* Acknowledging the difficulties of achieving interprofessional collaboration, WHO recommends that IPE is fostered already at the undergraduate level [4]. It is encouraging to see how research into the effects of undergraduate IPE has found increasingly positive attitudes toward members from different professional groups [7, 13–15], improved role clarity [3, 16], and enhanced teamwork skills [1, 2, 13, 17]. However, in our search for effects of IPE in mental health, we found limited evidence to substantiate the benefits of IPE interventions. For example, the 16 studies identified in Pauzé et al.’s (2010) systematic review of IPE programs for postgraduate mental health staff reveal a lack of rigorous studies of the effects of IPE in mental health education [3]. There is growing evidence to suggest that undergraduate IPE has positive contributions to professional practice as well as to clinical outcomes [8]. With the continuous growth in IPE activities, we found that a systematic review of studies of mental health education was timely, not least to provide a synthesis of the best available evidence for recommendations for future undergraduate IPE interventions. Our search strategy was based on the PRISMA guideline with regard to participants, interventions, comparisons, outcomes, and study designs [18]. We present a systematic review of studies describing the effects of IPE interventions on undergraduate healthcare students’ educational outcomes, compared to those of conventional clinical training in mental health.

## Methods

The review is structured in accordance with the Population, Intervention, Comparison, Outcome, and Study (PICOS) design framework [18], which was also used for the identification of key concepts for an effective search strategy. The electronic databases MEDLINE, CINAHL, PsychINFO, and EMBASE were searched. Our search terms were identified in collaboration with a research librarian in order to specifically address the aim of our review. Combinations of the following search terms were used: undergraduate, inter/multi-professional education, inter/multi-disciplinary education, mental health, and educational outcomes. The keywords were used in each electronic database to identify all types of IPE interventions in mental health education at the undergraduate level. We searched among papers published between January 2001 and August 2017 in English, German, or one of the Scandinavian languages. The studies present clinical IPE interventions with specific educational outcomes, preferably with a comparison group. We included only studies involving undergraduate students undertaking clinical training in mental health from the following professions: medicine, nursing, occupational therapy, pharmacy, physiotherapy, psychology, and social work. The mental health criterion was employed to identify adults (aged 18 years and over) with any form of mental health problem, except those relating to a primary diagnosis of learning disability, substance abuse, or dementia. Further information can be provided to readers by request.

Freeth et al. [19] reclassified Kirkpatrick’s [20] typology of educational outcomes from four to six outcomes of IPE was incorporated into the review to ensure a focused and unambiguous description of outcomes. The six levels of the model are outlined below:

- Level 1—Reaction: learners’ general views of and perspectives on the learning experience, its presentation, content, teaching methods, and the quality of teaching
- Level 2a—Attitudes/perceptions: outcomes related to changes in interprofessional attitudes or perceptions among participant groups, toward patients and their conditions, care, and treatment
- Level 2b—Knowledge/skills: knowledge relates to the acquisition of concepts, procedures, and principles of interprofessional collaboration. Skills relate to problem-solving and social skills relevant to collaboration
- Level 3—Behavioral change: measurements relate to changes of behavior in the workplace
- Level 4a—Organizational change: in relation to major changes in organizational policies or clinical pathways to promote interprofessional collaboration and communication
- Level 4b—Benefits to patients: improvements in the health and well-being of patients as a direct result of an IPE program. Such improvements include results of health status measures, duration of hospital stay, complication rates, readmission rates, patient satisfaction, continuity of care, and costs

### Searching, Reviewing, and Abstracting

**Fig. 1:**
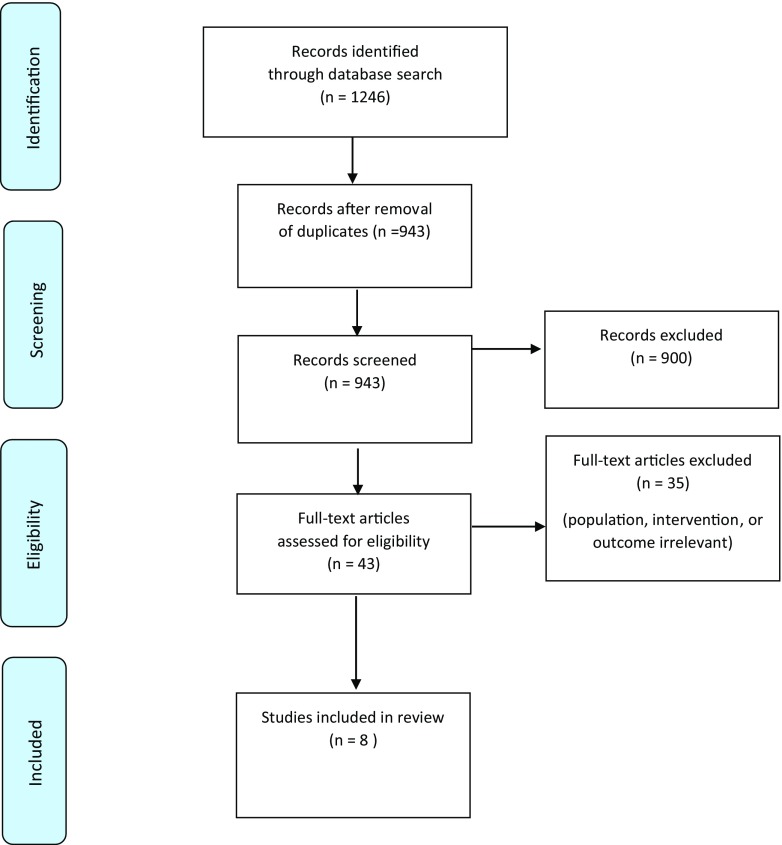
PRISMA four-phase flow diagram of study selection [21]

To capture the largest possible number of abstracts, we incorporated very broad search terms in the initial search of the four databases. The initial yield of 1246 titles was reduced to 943 after duplicates were removed. Our screening of the articles’ abstracts decimated their number to 43. The full texts of the 43 articles were then reviewed to assess their match with the selection criteria, leaving eight papers for inclusion in the study. Abstract screening and full-text reading to identify any which reported the use of IPE was done by the first author (MM). The included papers were reviewed by two authors (MM and SA) to ensure they met the agreed selection criteria. Problem over agreeing papers would be resolved by a third person (BN). Figure 1 provides an overview of the literature search process in a PRISMA four-phase flow diagram [21].

### Quality Assessment

The evidence presented in the eight papers was assessed according to methodological approach, description of learner outcomes, and evaluation of the overall quality of the data reported. The methodological quality of each paper was judged by several criteria. Our assessment took into account the strength of the research design, whether specific outcomes were reported, the methods employed for data collection, and the sampling of participants. This process involved an evaluation of the presented evidence in relation to methodology and educational outcomes as well as the clarity of the presented results. The following guidelines were used in assessing the studies: (a) Critical Appraisal Skills Program (CASP) to evaluate the qualitative and cohort studies [22]; (b) the Mixed Methods Appraisal Tool (MMAT) to evaluate the mixed method studies [23]. In addition, the risk of bias in each study was judged using the Risk of Bias in Non-randomized Studies tool – of Interventions (ROBINS–I) [24]. Following Pauzé (2010), the reviewed papers were assigned to one of the following categories of evidence quality: “good,” “acceptable,” “poor,” or “unacceptable.”

## Results

Our findings are presented in three sections dealing with (a) the characteristics of the included studies, the details given of the IPE interventions, and the study results; (b) the studies’ description of methodology and key information relating to the reported outcomes (PICOS); and (c) methodological considerations relating to the overall quality of the included studies.

### Study Characteristics

Table 1 gives an overview of the characteristics and results of the included IPE studies, including their design, reported outcomes, intervention, duration of intervention, studied profession, and data collection.

**Table 1:** Study characteristics and results of individual studies

Study	Aim	Research design	Outcomes and measures	Reported outcomes according to Kirkpatrick	Type of intervention	Duration	Type of students	Data collection
Barnes et al. (2006)	To improve interprofessional skills; and to increase awareness of the working from a user’s perspective	Longitudinal, B/A with control group	To assess learners’ reactions to user-trainers as course members; changes in knowledge and skills; and changes in individual and organizational practice; quality of care; social function and quality of life	Attitudes; knowledge/skills; behavioral; practice; patients (2a, 2b, 3, 4a, 4b)	Lectures by professional and service users, partnership with service users	1 day/week, 2 years	Nursing, social work, occupational therapy, psychology, medicine	Observation, interview, questionnaires
Barnett et al. (2015)	To investigate networking; collaboration and practiced supported student learning; organization’s strengths and opportunities regarding IPE and learning	Mixed methods	Readiness for interprofessional learning surveys (RIPLS); social network survey; role clarification activity; observation: participants working through a clinical case study	Attitudes, behavioral (2a, 3)	Workshops	2–3-h workshop	Nursing, social work, occupational therapist, psychology	Observation, interview, questionnaires
Curran et al. (2012)	To integrate IPE in collaborative mental health practice across the pre- and postlicensure continuum of education	Longitudinal, B/A	Participant satisfaction; attitudes toward teamwork; team work abilities; (quality of care)	Attitudes, knowledge/skills (2a, 2b)	Workshops, introduction to standardized patients (SP)	2 days	Nursing, allied health, medicine	Focus groups, interviews, questionnaires
Furness et al. (2011)	To develop sustainable models of IPE which would promote and facilitate the professional skills of students through collaborative working within the practice setting	Multiple case study	To evaluate two subsequent interprofessional practical learning sites. Questions were based on learners’ reaction; behavior change; facilitator role; impact upon practice	Attitudes, behavioral, practice, patients (2a, 3, 4a)	Presentation of service user “stories”, PBL sessions regarding IPE, workshops with service users and relatives	4 weeks	Nursing, medicine, social work, occupational therapy	Focus groups, interviews
Kinnair et al. (2012)	To establish an existing interprofessional educational framework (the Leicester Model) into mental health practice (to undergraduates) in order to improve care	Mixed methods	To assess course-specific learning outcomes; attitudes; interprofessional patient-centered learning/knowledge; team working; role clarity; practice and facilitators’ role	Attitudes, knowledge/skills, practice (2a, 2b, 4a)	CPA assessment with user perspective, shared reflection, feedback presentation, group discussion	1 year	Medicine, nursing, social work, occupational therapy, pharmacy	Focus groups, interviews, questionnaires
Priest et al. (2008)	To explore interprofessional attitudes arising from shared learning in mental health education (undergraduate level)	Longitudinal, B/A	To assess change over time in knowledge; awareness of interprofessional mental health; change in interprofessional attitudes; role clarity; team working (RIPLS)	Attitudes, knowledge/skills (2a, 2b)	IPE sessions with group activities and problem-based learning (PBL) with clinical vignettes	2 years	Nursing, psychology	Questionnaires
Reeves et al. (2006)	To enhance collaborative practice in mental health teams and to explore the usefulness of the presage-process-product (3P) framework for analysis	Mixed methods, B/A	To assess perception of collaboration and roles; interprofessional knowledge and skills; reflection	Attitudes, knowledge/skills (2a, 2b)	IPE workshops, team discussion, shared reflection	3-, 2-h workshop	Medicine, social work, nursing, occupational therapy	Observation, focus group, questionnaires
Rolls et al. (2002)	To promote collaboration and to establish and 40-day interprofessional course in mental health practice	Mixed methods	To assess course-specific knowledge; interprofessional skills; attitudes toward other professions	Attitudes, knowledge/skills (2a, 2b)	Modules on assessment, case management, and psychosocial interventions	40 days	Nursing, psychology, occupational therapy, social work	Interview, case study, questionnaires

*B/A*, before/after; *IPE*, interprofessional education; *CPA*, care program approach; *CASP*, Critical Appraisal Skills Program; *MMAT*, Mixed Methods Appraisal Tool

### Methodological Description

#### Population

The studied IPE interventions targeted a range of healthcare students from either medicine, nursing, occupational therapy, physiotherapy, psychology, or social work. The number of students involved in each intervention ranged from 19 [25] to 300 [1]. Between two and five different healthcare professions were represented in the interventions.

Five of the studies were published between 2008 and 2016. The preponderance of publications from this period may reflect the WHO’s repeated calls for improved collaboration among mental healthcare professionals and governmental policies over the preceding 10 years [4]. The same period also saw increasing evidence that collaborative mental healthcare is capable of improving the quality of services [3–5, 9].

#### Intervention

The included studies all concerned undergraduate clinical education in mental health. The duration of the IPE interventions varied from 1 day to training sessions conducted over a month. The least extensive element lasted 6 h over 2 days [26], while the most extensive program appeared to be 40 days [27].

A variety of small group learning activities were reported, including group discussions [27], workshops [7, 13, 26], standardized patients [13], problem-based learning to enhance collaboration [1, 13, 14], and reflection [7]. Reeves et al. employed team discussion and shared reflection to enhance knowledge of selected issues related to effective collaboration and communication [7]. A few studies used didactic formats with service users [17, 28]. In their programs, Barnes et al. allotted service users a role as co-students as well as a management and teaching role. This had the dual aim of improving students’ interprofessional skills and raising their awareness of the importance of working from a service user’s perspective [17].

#### Outcomes

All but one study reported positive outcomes related to the studied IPE intervention; only Rolls et al. [25] failed to clearly report their results. Eight studies reported *attitudes* toward other professions (level 2a in Kirkpatrick’s models of evaluation). Six studies reported change in *knowledge* and *skills* [1, 7, 13, 14, 17, 26]; three studies reported *behavior* changes that enhanced collaboration [17, 25, 27]. Changes in *organizational practice* [1, 17, 27] were reported in three studies, while only two reported outcomes related to patients’ benefit [17, 27]. A description of outcomes and associated measures assessed in each study and summary of learning outcomes’ impact is presented in Tables 1 and 2.

#### Study Design

Four of the studies used a before and after design; three with longitudinal, before and after design [13, 14, 17]; one also with a control group in a comparable setting where no interprofessional training had taken place [17]. A cross-sectional approach was used in one study [26], while another was a case study [27].

Questionnaires were the principal method of data collection used in seven studies. Questionnaires, interviews, and observations were the most common techniques used by eight, five, and three of the studies, respectively. Other methods included focus group interviews and case studies (see Table 2).

**Table 2:** Summary of learning outcomes

Outcome (Kirkpatrick’s levels)	Impact
2a. Attitudes/perceptions	• More positive attitudes toward collaboration with patients [1, 13, 14, 17, 24, 26]
2b. Knowledge/skills	• Improved role clarity and individual authority [1, 7, 13, 14, 17]
3. Behavioral change	• Increase in shared decision-making [17, 24]
4a. Changes in organizational practice	• Involving users in decision-making [17, 25] • Use of practice guidelines [1] • Involving users in teaching [1]
4b. Benefits to patients	• Improved social functioning and life satisfaction [17]

### Methodological Considerations

#### Bias Risk

In general, methodological issues were insufficiently discussed in the reviewed studies. Details regarding study limitations and data collection methods were sparse, with only three studies providing clear information on limitations [1, 13, 14]. Reported sources of bias related to the following: selection and detection [1, 13, 14], lack of comparison group [1, 13], findings primarily based on self-report [13], and dropout [14]. The risk of bias varied considerably across studies, four of which are judged to be at moderate risk [1, 7, 13, 17], as they provide sound evidence and few aspects prone to bias risk, such as the selection of the participants.

Two studies presented a serious risk of bias [25, 27], with problems concerning selection bias and insufficient information for several key areas. Caution should thus be taken with Rolls et al.’s [26] findings, as their study was judged to be at critical risk of bias.

As already mentioned, the overall quality of the evidence reported by the articles was determined by methodology, educational outcomes, and the clarity of the results presented.

Only Barnes et al.’s study [17] was considered of “good quality.” Its rigorous research design (longitudinal, before and after design with control group) and the complexity of the assessment of educational outcome levels (e.g., levels 2, 3, and 4) stood out. In addition, the studied IPE interventions were concisely described, had clear learning objectives, and the reported interventions lasted more than a year.

Six studies provided an “acceptable quality” of evidence, viz. [1, 7, 13, 14, 25, 27]. While appropriate research designs were used, with logical progression from methods to outcomes, their discussion of methodological issues was insufficient. In addition, the studied IPE interventions were significantly shorter in duration.

The study by Rolls et al. [26] was considered to be of “poor quality,” primarily due to its unclear reporting of sample size and incomplete description of evaluation methods and outcomes. Its weak design was indicated by the absence of baseline data collection, which precludes a convincing account of change relating to the IPE interventions. A summary of the assessment of the included studies is presented in Table 3.

**Table 3:** Summary of quality assessment (a synthesis of CASP/MMAT checklists + Risk of Bias)

Study	Clear research question?	Collected data address the research question?	Appropriate research design?	Recruitment strategy appropriate?	Measurements appropriate?	Outcome accurately measured?	Clear statement of findings?	Appropriate consideration given to limitations?	Risk of Bias (ROBINS-I)	Quality of evidence: overall rating
Barnes et al. (2006)	Yes	Yes	Yes	Yes	Yes	Yes	Yes	Cannot tell	Moderate risk of bias	Good quality
Barnett et al. (2015)	Yes	Cannot tell	No	Cannot tell	Yes	Cannot tell	Yes	No	Serious risk of bias	Acceptable–poor quality
Curran et al. (2012)	Yes	Yes	Yes	Yes	Yes	Cannot tell	Cannot tell	Yes	Moderate risk	Acceptable quality
Furness et al. (2011)	Yes	Yes	No	Yes	Yes	Yes	Yes	Cannot tell	Serious risk of bias	Acceptable quality
Kinnair et al. (2012)	Yes	Yes	Yes	Cannot tell	Yes	Cannot tell	Cannot tell	Yes	Moderate risk of bias	Acceptable quality
Priest et al. (2008)	Yes	Cannot tell	Yes	Cannot tell	Yes	Cannot tell	Yes	Yes	Moderate or serious risk of bias	Acceptable quality
Reeves et al. (2006)	Yes	Yes	Yes	Yes	Yes	Cannot tell	Cannot tell	Cannot tell	Moderate risk of bias	Acceptable quality
Rolls et al. (2002)	Yes	Cannot tell	No	Cannot tell	Yes	Cannot tell	Cannot tell	Cannot tell	Critical risk of bias	Poor quality

*CASP*, Critical Appraisal Skills Program; *MMAT*, Mixed Methods Appraisal Tool; *ROBINS–I*, Risk of Bias in Non-randomized Studies of Interventions

## Discussion

The included studies showed great variation with respect to the IPE interventions examined and assessment methods. The ambiguous results of the eight different IPE interventions, undertaken in eight different clinical settings, are thus unremarkable. The quality of the studies furthermore varied considerably. One study had a robust longitudinal, before and after design, that included a control group [17]; six studies demonstrated adequate alignment between the objectives of the study and the reported outcomes, although their research designs were less rigorously described [1, 7, 13, 14, 25, 27].

Despite this heterogeneity, we found evidence that students of mental health responded well to IPE, especially in terms of more positive attitudes toward the contribution of other professions [1, 7, 13, 14, 17, 25] and increased knowledge of and skills in collaboration [1, 7, 13, 14, 17, 26]. However, we found no substantial evidence of changes in behavior or organizational practices, which possibly reflects the complexity of IPE interventions and attitudinal differences toward IPE stemming from differences in work culture, as has also been found by other review studies [3, 28–30].

The apparent lack of association between undergraduate mental health IPE interventions and behavioral change corresponds with findings from other studies that outcomes tend to be discernible only at Kirkpatrick’s levels 1 (learners’ reaction), 2a (attitudes), and 2b (knowledge and skills) [3, 7, 10, 27]. Thus, only two of the eight studies reviewed here [17, 27] reported outcomes related to patient care. Similarly, the literature in general reveals a lack of involvement of users in the undergraduate IPE interventions [1, 10, 28]. Such a patient-centered approach could be ensured by involving patients in the planning, delivery, and evaluation of IPE interventions [16, 17].

Two of the reviewed studies reported students’ appreciation of the rare opportunity to learn directly from users [17, 27]. Reeves and Pauzé [3, 7] emphasize undergraduate learners’ great benefit from the inclusion of users in IPE. Service user involvement in education was crucial to students’ positive perceptions [27]. However, the traditional teacher and student relationship may be challenged by user participation, as indicated by several studies [1, 9, 17, 26, 27]. Some students feel unable to openly discuss questions or challenge professionals, or express criticism of users’ views [17]. Conversely, another study highlights that preparation and support are particularly important for vulnerable mental health service users as they felt uneasy and tense telling their story to the students [1, 27].

Although only two of the included studies explicitly reported improvement in patients’ conditions resulting from IPE [17, 27], it seems to be a reasonable conjecture that changes in students’ behavior and organizational practices may have positively impacted patients. However, the effects of IPE remain unclear without direct evidence from patients’ care. The reviewed studies moreover exhibit a number of shortfalls, such as insufficiencies in the reporting of methods and discussion of limitations [25–27], uncertainty as to whether the initial effects of IPE were maintained over time [1, 25–27], and poor descriptions of the evaluated IPE interventions [25, 26].

The use of questionnaires for data collection ensures the recording of outcomes but precludes the obtainment of process measures, a shortfall that may have been resolved by collecting observational data. Three studies [7, 17, 25] thus provided a more robust understanding of processes and outcome data by combination of methods.

Baseline activities and longitudinal study of the IPE students were reported only in three studies [13, 14, 17]. For future study, research designs which include multi-method and longitudinal dimensions in order to understand both the processes and the impact of undergraduate IPE would be pertinent. For example, the initial impact of IPE is likely to diminish over time, especially where continued input to consolidate learning is absent.

The findings of the studies reviewed here suggest that, in comparison with standard clinical training, IPE in mental healthcare may improve educational outcomes, for example with regard to attitudes toward other professions and interprofessional skills.

Despite our adherence to PRISMA guidelines for systematic reviews, the findings of this study are limited by the selection of search terms and databases. Moreover, only studies published in an English, German, or Scandinavian language were included. As a result, potentially relevant IPE studies may have been excluded. We acknowledge the risk of publication bias, which may mean that studies reporting negative outcomes were not published and that such outcomes are underreported in the present studies [31].

Although the findings of this review corroborate those of Pauzé et al.’s [3], the heterogeneity of IPE interventions, study designs, and outcomes preclude us from offering unambiguous conclusions and recommendations regarding the effect of IPE in mental healthcare.

Except for one study of good quality, the strength of evidence presented by the studies is found to range from acceptable to poor. Future research would benefit from using a limited set of validated and reliable tools for the assessment of attitudes, knowledge, behavior, and organizational practices. Finally, in order that substantial evidence of undergraduate IPE in mental health can be provided, both the number and quality of studies need to increase.

### Recommendations for Future Interventions

Based on our review, we recommend establishing preconditions for undergraduate IPE, and ensuring appropriate support, design, and evaluation of IPE interventions.

Although our recommendations target undergraduate IPE in mental healthcare, their general nature ensures their relevance for IPE throughout the healthcare services.

In summary, our systematic literature search identified eight studies of undergraduate IPE in mental health. The evaluation of the studies revealed inadequacies in the description of methods and incomplete information about the interventions. In a situation where policymakers continue to press for the adoption of IPE in mental healthcare, there is an urgent need to remedy the lack of evidence into the effects of undergraduate IPE in mental health. The uncertainties regarding the impact of undergraduate IPE in mental health include the processes and the long-term impacts of IPE in mental health services. The lack of higher quality papers and the diversity of methodologies in the selected sample may suggest the need for further research. Finally, we recommend that service users are involved in the planning, implementation, and evaluation of future undergraduate IPE interventions in mental health.
